# Supplementary material for: The OsSGS3-tasiRNA-OsARF3 module orchestrates abiotic-biotic stress response trade-off in rice
Source: Nat Commun. 2023 Jul 24;14:4441. doi: 10.1038/s41467-023-40176-2 (PMC10366173; doi:10.1038/s41467-023-40176-2)
Supplement: Supplementary file 1 — Supplementary Information [file 41467_2023_40176_MOESM1_ESM.pdf]

SUPPLEMENTARY INFORMATION FOR

The OsSGS3-tasiRNA-OsARF3 module orchestrates abiotic-biotic stress response trade-off in rice

Xueting Gu[1,8], Fuyan Si[2,8], Zhengxiang Feng[3,8], Shunjie Li[3,8], Di Liang[1,8], Pei Yang[3], Chao Yang[2], Bin Yan[2], Jun Tang[1], Yu Yang[3], Tai Li[3], Lin Li[3], Jinling Zhou[3], Ji Li[2], Lili Feng[1], Ji-Yun Liu[1], Yuanzhu Yang[4], Yiwen Deng[1], Xu Na Wu[3], Zhigang Zhao[5], Jianmin Wan[5], Xiaofeng Cao[2,6,7], Xianwei Song[2,*], Zuhua He[1,*] and Junzhong Liu[3,*]

[1]National Key Laboratory of Plant Molecular Genetics, CAS Center for Excellence in Molecular Plant Sciences, Institute of Plant Physiology and Ecology, Chinese Academy of Sciences, Shanghai 200032, China.

[2]State Key Laboratory of Plant Genomics and National Center for Plant Gene Research, Institute of Genetics and Developmental Biology, Chinese Academy of Sciences, Beijing 100101, China.

[3]Center for Life Sciences, School of Life Sciences, State Key Laboratory of Conservation and Utilization of Bio-Resources in Yunnan, Yunnan University, Kunming 650500, China.

[4]Department of Rice Breeding, Hunan Yahua Seed Scientific Research Institute, Changsha, Hunan 410119, China.

[5]National Key Laboratory for Crop Genetics and Germplasm Enhancement, Nanjing Agricultural University, Nanjing 210095, China.

[6]University of Chinese Academy of Sciences, Beijing 100039, China.

[7]CAS Center for Excellence in Molecular Plant Sciences, Chinese Academy of Sciences, Beijing 100101, China.

[8]These authors contributed equally: Xueting Gu, Fuyan Si, Zhengxiang Feng, Shunjie Li, Di Liang.

[*]Correspondence:

xwsong@genetics.ac.cn; zhhe@cemps.ac.cn; liujunzhong@ynu.edu.cn

Supplementary Figures: 1-14

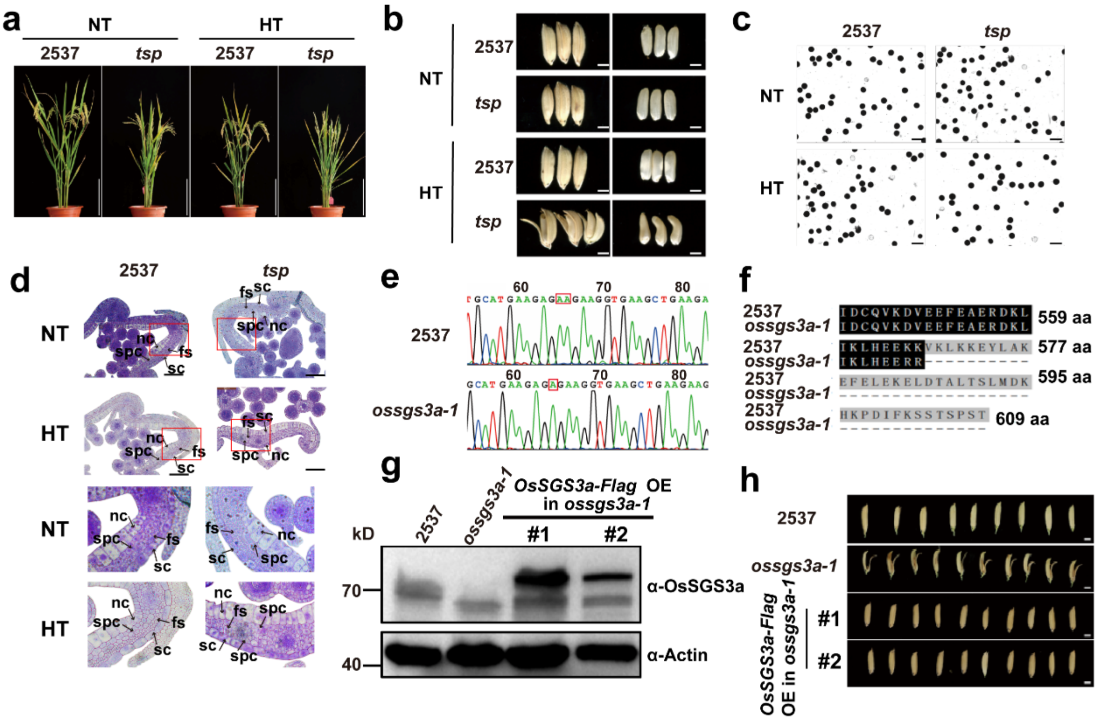

Supplementary Fig. 1 Phenotypes and genotypes of the *tsp* mutant and 2537.

a Plants of 2537 and *tsp* grown at normal (NT)/high field temperature (HT). Scale bars, 20 cm. **b** Grain development in 2537 and *tsp* plants grown under normal/high temperature. Scale bars, 3 mm. **c** The pollen viability of 2537 and *tsp* plants grown under normal/high temperature as determined by I_2-KI staining. Scale bars, 50 μm. **d** Semithin sections of palea in 2537 and *tsp* plants grown under normal/high field temperature. Silicified cells (sc), fibrous sclerenchyma (fs), spongy parenchymatous cells (spc), nonsilicified cells (nc). Scale bars, 100 μm. Plants were grown in Shanghai in the summer with high field temperature (HT, ⩾35°C) frequently occurring at the rice booting stage, or in Hainan in the winter with normal temperature (NT) suitable for rice growth (**a-d**). **e** The schematic of the mutation of *OsSGS3a* in *ossgs3a-1* mutant. **f** The alignment of OsSGS3a protein sequences in 2537 and *ossgs3a-1*. **g** Western blotting analysis of OsSGS3a protein levels using anti-OsSGS3a antibody in the florets of 2537, *ossgs3a-1* and two complementary lines grown under high field temperature. Equal protein loading was confirmed with the anti-OsActin antibody. Similar results were obtained from three independent experiments (**c**, **d**, **g**). Source data are provided as a Source Data file. **h** Seeds of 2537, *ossgs3a-1* and two complementation lines grown under high field temperature. Scale bars, 3 mm.

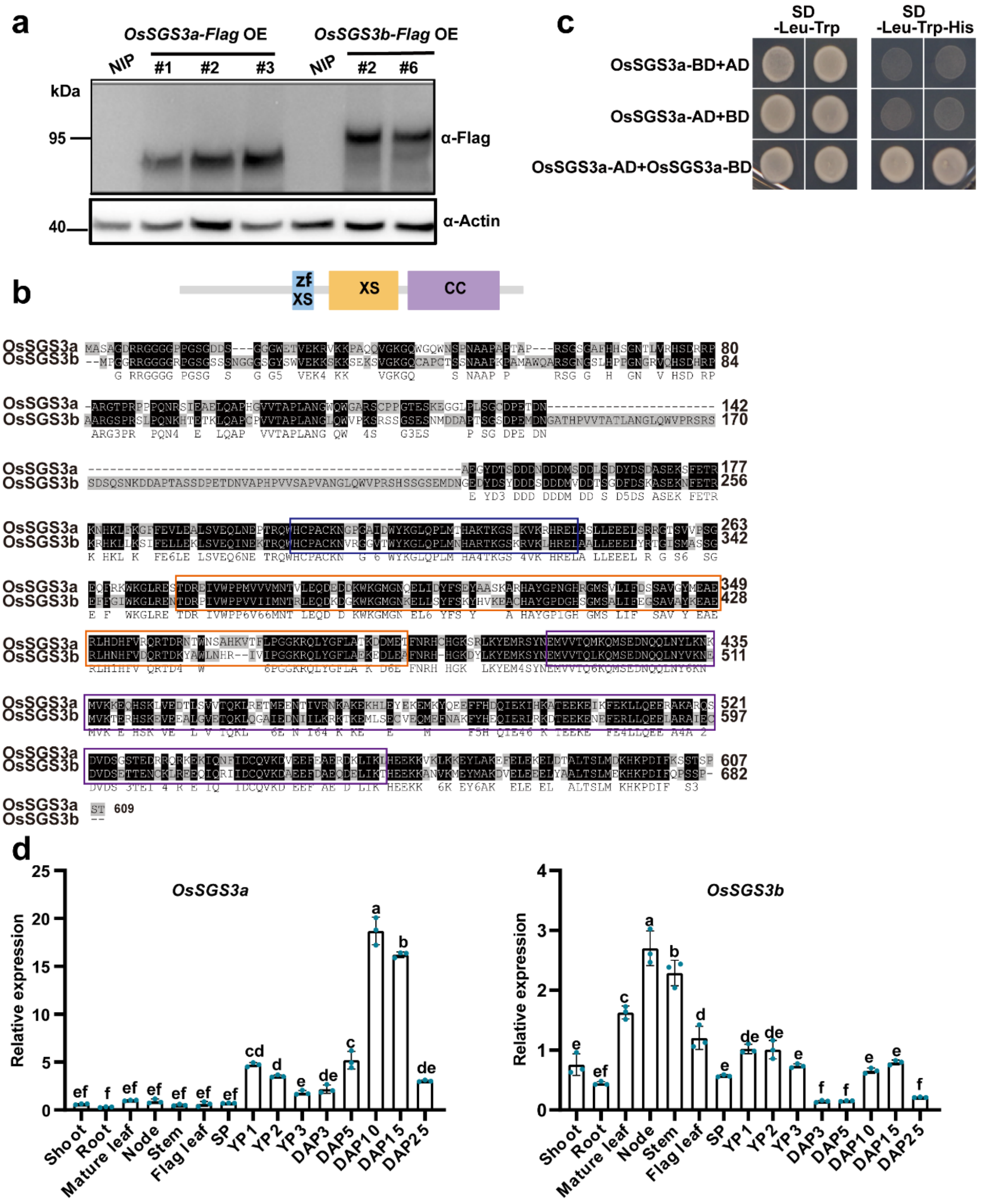

Supplementary Fig. 2 The characteristics of OsSGS3a and OsSGS3b.

a Immunoblot analysis of OsSGS3a-Flag and OsSGS3b-Flag protein abundance. OsActin served as a loading control. NIP was used as a negative control. **b** Alignment of OsSGS3a and OsSGS3b protein sequences. The zf-XS, XS and CC domains were indicated. **c** OsSGS3a protein interacts with itself in yeast. **d** Levels of *OsSGS3a* and *OsSGS3b* RNA as measured by qRT-PCR in different samples from plants grown in the field. Data are presented as means ± s.d. (*n* = 3, biologically independent samples). *OsActin* served as an internal control to normalize the expression of individual genes. Shoot, shoot of 4-week-old seedling; Root, root of 4-week-old seedling; Mature leaf, mature leaf of 8-week-old plant; Node, the first node of 8-week-old plant; Stem, the first stem of 8-week-old plant; SP, spikelet; YP1, 1-3 cm young

panicle; YP2, 3-5 cm young panicle; YP3, 5-10 cm young panicle; DAP, days after pollination. Different letters indicate significant differences according to one-way ANOVA with Tukey's HSD post hoc analysis ($P < 0.05$). Exact P values are provided in Supplementary Data 7. Source data are provided as a Source Data file (**a**, **d**). Similar results were obtained from two independent experiments (**a**, **c**, **d**).

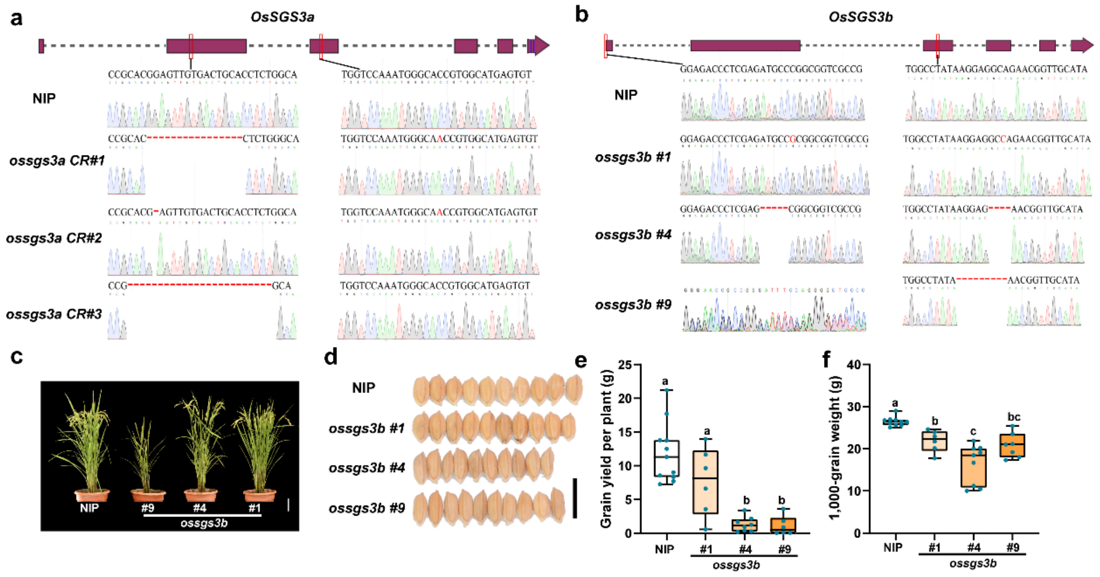

Supplementary Fig. 3 The phenotypes of *ossgs3a CR* and *ossgs3b* mutants.

a Schematic of the mutation of *OsSGS3a* in positive transformed calli expressing *OsSGS3a* CRISPR constructs. **b** Schematic of the mutation of *OsSGS3b* in *ossgs3b* mutants. **c**, **d** Plants (**c**) and grains (**d**) of NIP and *ossgs3b* mutants grown under high field temperature. Scale bars, 10 cm (**c**) or 1 cm (**d**). **e**, **f** The grain yield per plant (**e**) and 1,000-grain weight (**f**) of NIP and *ossgs3b* mutants grown under high field temperature, shown as box plots ($n \geqslant 6$, biologically independent samples). Box plots show the median (central line) and interquartile range (IQR; from the 25th to 75th percentile); whiskers extend to minimum and maximum values within 1.5 times the IQR (**e**, **f**). Significant differences were determined by one-way ANOVA with Tukey's HSD post hoc analysis ($P < 0.05$). Exact P values are provided in Supplementary Data 7. Source data are provided as a Source Data file.

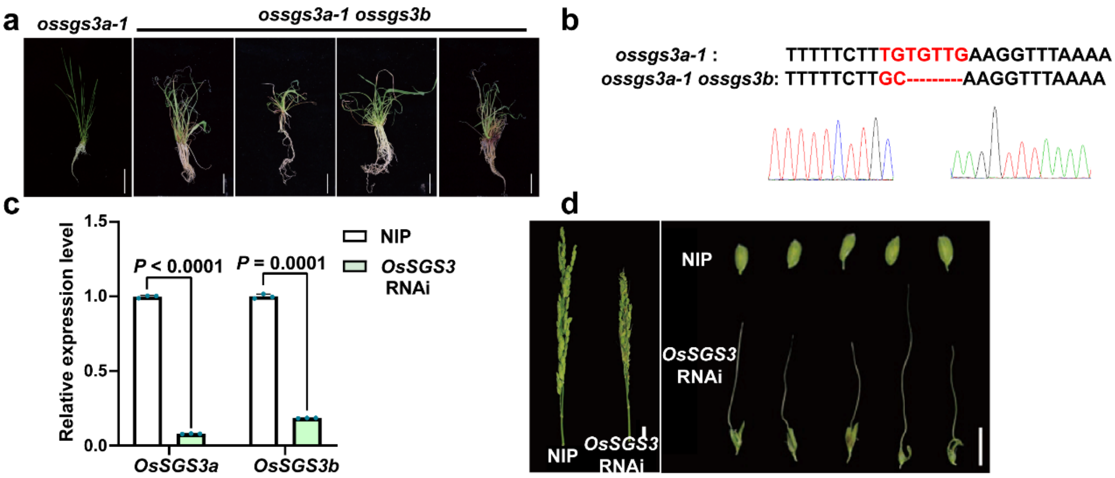

Supplementary Fig. 4 The phenotypes of *ossgs3a-1 ossgs3b* mutants and *OsSGS3* RNAi transgenic plants.

a Seedlings of *ossgs3a-1* and *ossgs3a-1 ossgs3b* mutants grown in a growth chamber at 28°C. Scale bars, 5 cm. **b** Schematic of the mutation of *OsSGS3b* in *ossgs3a-1 ossgs3b* mutants. **c** qRT-PCR analysis of *OsSGS3a/b* transcript levels in *OsSGS3* RNAi transgenic plants. *OsActin* served as an internal control. Data are presented as means ± s.d. ($n = 3$, biologically independent samples). Significant differences were determined by two-tailed Student's t-test. Source data are provided as a Source Data file. Similar results were obtained from two independent experiments. **d** Panicles (left) and seeds (right) of Nip and *OsSGS3* RNAi transgenic plants grown under high field temperature. Scale bars, 1 cm.

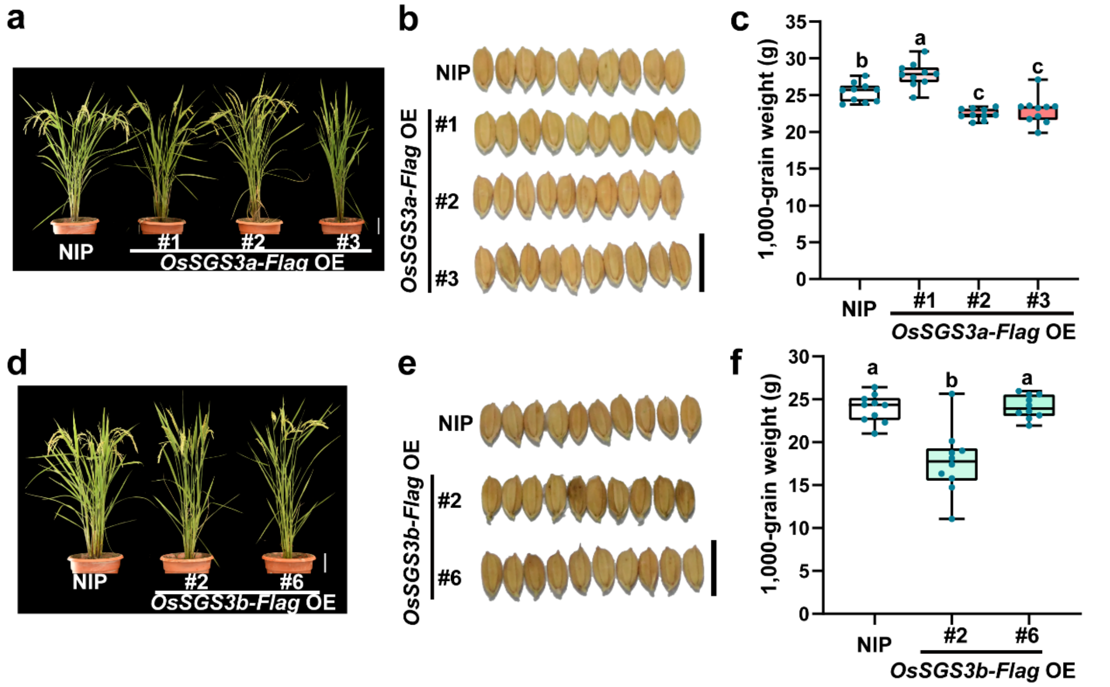

Supplementary Fig. 5 Overexpression of *OsSGS3a-Flag* or *OsSGS3b-Flag* did not increase 1,000-grain weight.

a Plants of NIP and three independent *OsSGS3a-Flag* transgenic lines. **b**, **c** Grains (**b**) and 1,000-grain weight (**c**) of NIP and *OsSGS3a-Flag* transgenic lines, shown as box plots (n = 10, biologically independent samples). **d** Plants of NIP and two independent *OsSGS3b-Flag* transgenic lines. **e**, **f** Grains (**e**) and 1,000-grain weight (**f**) of NIP and *OsSGS3b-Flag* transgenic lines, shown as box plots (n = 10, biologically independent samples). Box plots show the median (central line) and interquartile range (IQR; from the 25th to 75th percentile); whiskers extend to minimum and maximum values within 1.5 times the IQR (**c**, **f**). Scale bars, 10 cm (**a**, **d**) or 1 cm (**b**, **e**). Different letters indicate significant differences according to one-way ANOVA with Tukey's HSD post hoc analysis ($P < 0.05$) (**c**, **f**). Exact P values are provided in Supplementary Data 7 (**c**, **f**). Source data are provided as a Source Data file (**c**, **f**). All plants were grown under high field temperature (**a-f**).

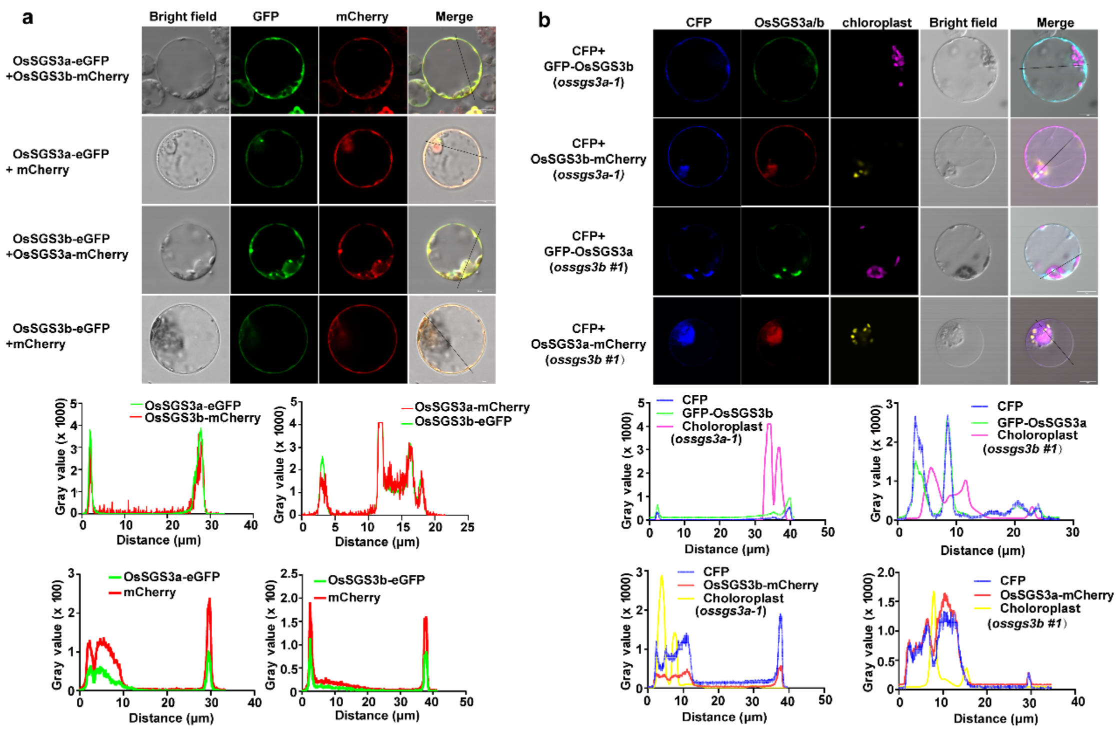

Supplementary Fig. 6 Subcellular localization of OsSGS3a and OsSGS3b proteins. **a** OsSGS3a and OsSGS3b were co-localized in the protoplast of NIP. NIP protoplasts were co-transformed with OsSGS3a-eGFP and OsSGS3b-mCherry, OsSGS3a-eGFP and mCherry, OsSGS3b-eGFP and OsSGS3a-mCherry, or OsSGS3b-eGFP and mCherry. Scale bars, 10 μm. **b** Representative localization images of GFP-OsSGS3b or OsSGS3b-mCherry transiently expressed in the *ossgs3a-1* protoplast (upper) and GFP-OsSGS3a or OsSGS3a-mCherry transiently expressed in the *ossgs3b* protoplast (lower). CFP served as a non-specific localized marker. Scale bars, 10 μm. The plotting of pixel intensities for the different color channels along transects was conducted (**a**, **b**). Experiments were repeated three times with similar results (**a**, **b**). Source data are provided as a Source Data file (**a**, **b**).

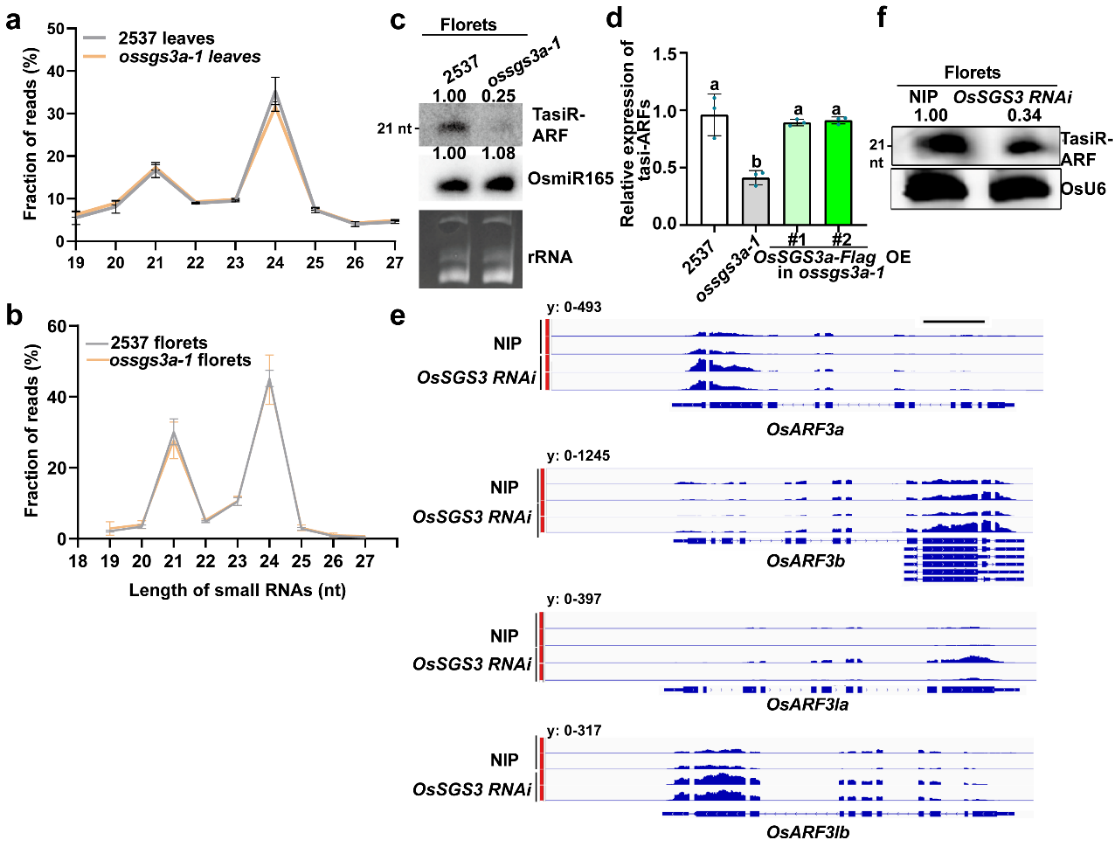

Supplementary Fig. 7 OsSGS3a modulates the biogenesis of tasiR-ARFs.

a, **b** Small RNA size distribution in leaves (**a**) or florets (**b**) of 2537 and *ossgs3a-1*. The abundance of small RNAs in each size class is presented as a percentage of the total abundance of 19~27-nt small RNAs. Leaves were collected from 2-week-old seedlings grown in the growth chamber at 28°C and florets were sampled from 2537 and *ossgs3a-1* grown under high field temperature. **c** Levels of tasiR-ARFs and OsmiR165 in the florets of 2537 and *ossgs3a-1*. The abundance of rRNA was used as a loading control. **d** Levels of tasiR-ARFs in the leaves of 2537, *ossgs3a-1*, and complementary transgenic plants grown in a growth chamber as determined by qRT-PCR. Values are mean ± s.d. ($n = 3$, biologically independent samples) (**a**, **b**, **d**). Different letters indicate significant differences according to one-way ANOVA with Tukey's HSD post hoc analysis ($P < 0.05$). Exact P values are provided in Supplementary Data 7. **e** Genome browser view of the *OsARF3la*, *OsARF3lb*, *OsARF3a*, and *OsARF3b* mRNAs in the leaves of NIP and *OsSGS3* RNAi grown in a growth chamber at 28°C. Scale bar, 1 kb. **f** Knockdown of OsSGS3 decreased the abundance of tasiR-ARFs in florets. *OsU6* was used as a loading control (**c**, **f**). The intensity of the blots was quantified (**c**, **f**). Experiments were repeated three times with similar results (**c**, **f**). Source data are provided as a Source Data file (**a-d**, **f**).

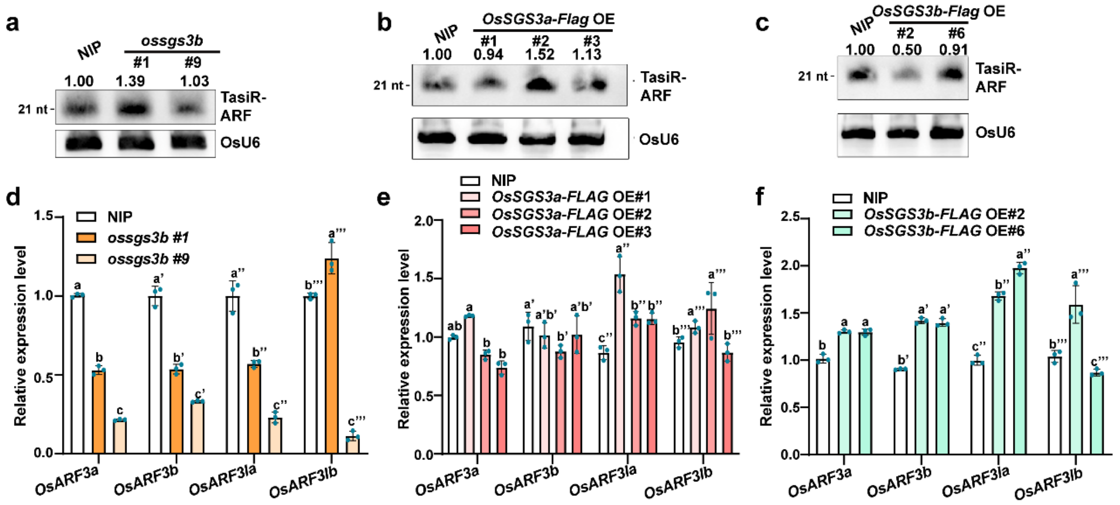

Supplementary Fig. 8 Loss-of-function of *OsSGS3b* or overexpression of *OsSGS3a-Flag* or *OsSGS3b-Flag* did not consistently modulate the abundance of tasiR-ARFs and the expression of *OsARF3a/b/la/lb* in leaves.

a-c RNA blot of tasiR-ARFs in the leaves of *ossgs3b* (**a**)*, OsSGS3a-Flag* OE (**b**) or *OsSGS3b-Flag* OE (**c**) seedlings grown in the field. *OsU6* was used as a loading control. The intensity of the blots was quantified. **d-f** qRT-PCR analysis of *OsARF3la*, *OsARF3lb*, *OsARF3a*, and *OsARF3b* mRNAs in the leaves of *ossgs3b* (**d**)*, OsSGS3a-Flag* OE (**e**) or *OsSGS3b-Flag* OE (**f**) transgenic plants grown in the field, respectively. *OsActin* served as an internal control (**d-f**). Values are mean ± s.d. (n = 3, biologically independent samples) (**d-f**). Different letters indicate significant differences according to one-way ANOVA with Tukey's HSD post hoc analysis ($P < 0.05$) (**d-f**). Exact P values are provided in Supplementary Data 7 (**d-f**). Source data are provided as a Source Data file (**a-f**). All experiments were repeated two times with similar results.

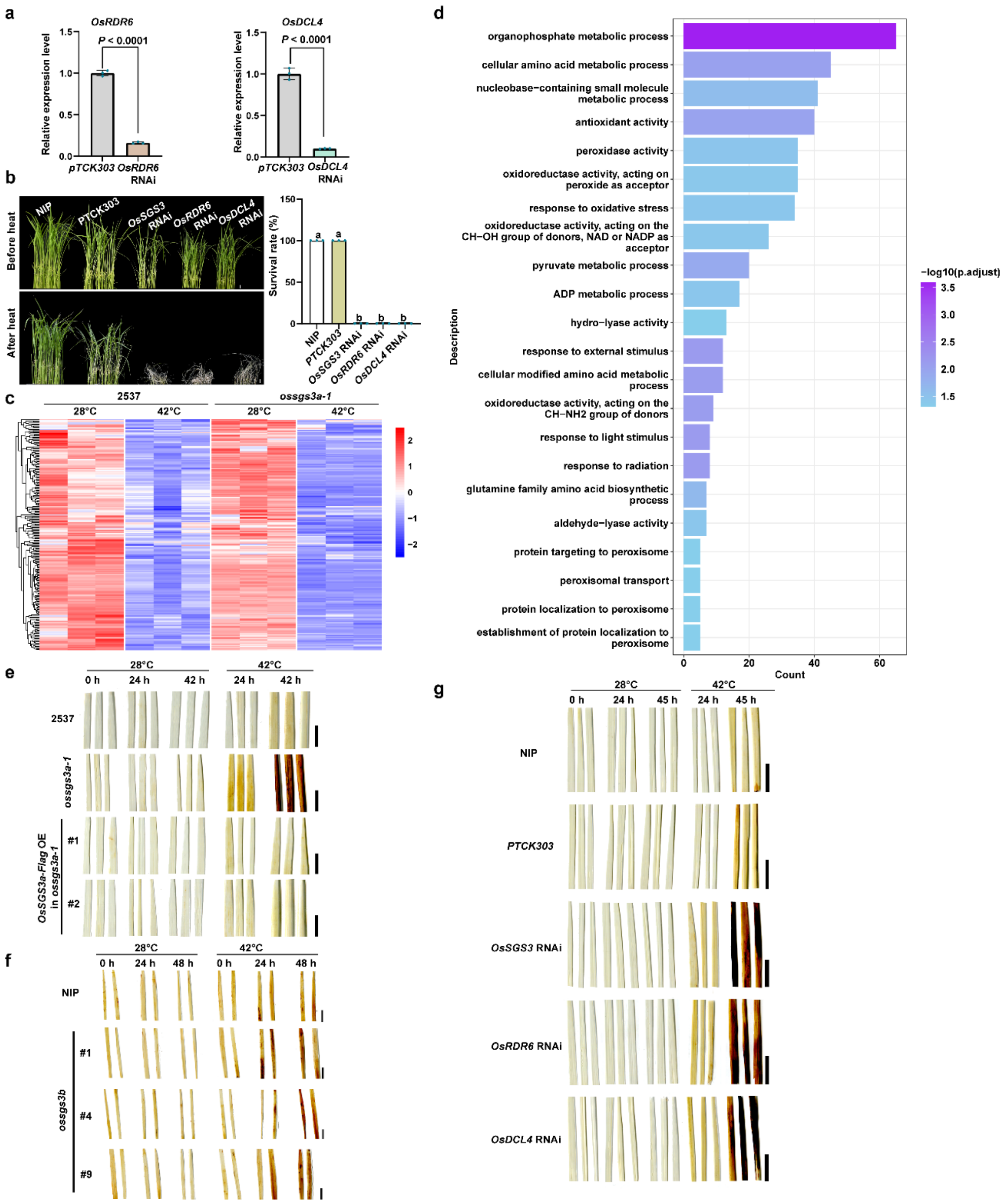

Supplementary Fig. 9 OsSGS3a/b-mediated thermotolerance involves ROS-related pathways.

a qRT-PCR analysis of *OsRDR6* and Os*DCL4* transcript levels in *OsRDR6* RNAi and *OsDCL4* RNAi transgenic plants grown in a growth chamber, respectively. *OsActin* served as an internal control. **b** Fewer *OsSGS3* RNAi, *OsRDR6* RNAi and *OsDCL4* RNAi plants survived compared with the wild-type and the PTCK303 when subjected to 42°C treatment. Values are mean ± s.d. (n = 3, biologically independent samples) (**a**, **b**). Significant differences were determined by two-tailed Student's t-test (**a**) or one-way ANOVA with Tukey's HSD post hoc analysis (P < 0.05) (**b**). Exact P values are indicated above the bars (**a**) or provided in Supplementary Data

7 (**b**). **c** Transcriptomic analysis of down-regulated genes in *ossgs3a-1* with respect to 2537 under heat stress. **d** Clustering and gene ontology (GO) analysis showed that down-regulated genes in (**c**) were enriched for reactive oxygen species (ROS)-related pathways. Source data are provided as a Source Data file (**a-d**). **e-g** The accumulation levels of H_2O_2 in the indicated plants as determined by DAB staining. 12-day-old seedlings were treated at 42°C for the indicated hours in a growth chamber before DAB staining. Scale bars, 1 cm (**b**, **e-g**). Experiments were repeated two times with similar results (**b**, **e-g**).

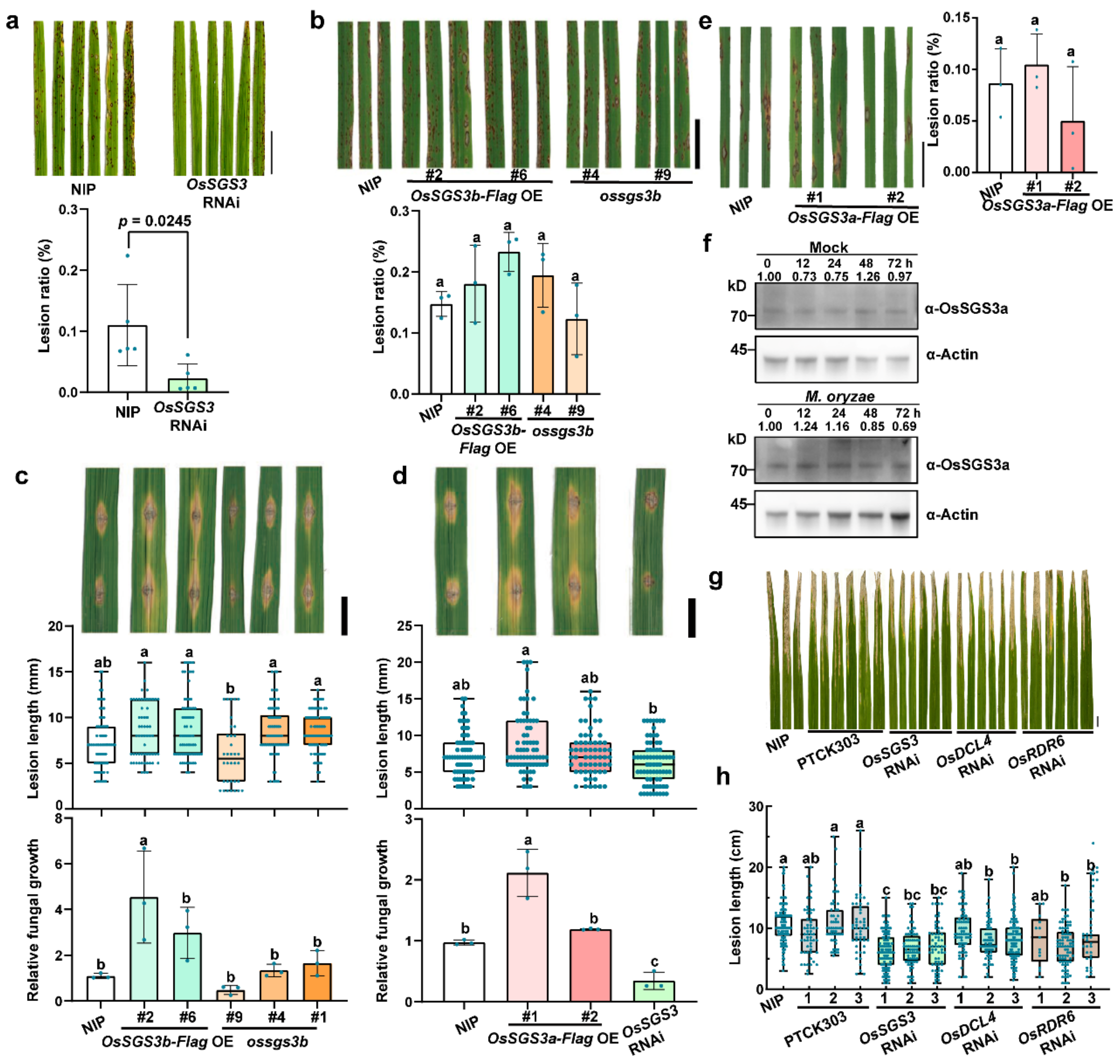

Supplementary Fig. 10 OsSGS3a negatively regulates rice resistance to *Xoo* and *M. oryzae*.

a, **b** Blast resistance of *OsSGS3* RNAi (**a**), *ossgs3b* and *OsSGS3b-Flag* OE (**b**) at 5 days post spraying inoculation with TH12. Lesion ratio (mean ± s.d.; *n* = number of biologically independent samples in the graph) were determined. **c**, **d** Blast resistance of *ossgs3b* and *OsSGS3b-Flag* OE (**c**), *OsSGS3* RNAi and *OsSGS3a-Flag* OE (**d**) at 7 dpi with punch injection inoculation (TH12). Similar wild-type NIP plants were used in **c**, **d**. Lesion length (mean ± s.d.; *n* = number of biologically independent samples in the graph) were determined. Relative fungal growth (mean ± s.d.; *n* = number of biologically independent samples in the graph) was determined with fungal *POT2* normalized to rice *Ubiquitin* by qRT-PCR. **e** Blast resistance of *OsSGS3a-Flag* OE at 5 days post spraying inoculation with TH12. Lesion ratio (mean + s.d.; *n* = 3 biologically independent samples) were determined. **f** Immunoblot analysis of OsSGS3a protein abundance during a time course of 0~72 h in the leaves of wild-type inoculated with *M. oryzae*. Two-week-old plants were infected with *M. oryzae*, and protein was extracted from infected and water mock control leaves collected at different infection time points. OsActin

served as a loading control. The intensity of the blots was quantified. **g**, **h** Bacteria blight resistance of *OsSGS3* RNAi, *OsDCL4* RNAi, and *OsRDR6* RNAi. Disease symptom (**g**) and lesion lengths (**h**) were measured at 14 dpi. Lesion lengths were shown as box plots ($n \geqslant 34$, biologically independent samples) (**h**). Box plots show the median (central line) and interquartile range (IQR; from the 25th to 75th percentile); whiskers extend to minimum and maximum values within 1.5 times the IQR (**c**, **d**, **h**). Significant differences were determined by two-tailed Student's *t*-test(**a**) or one-way ANOVA with Tukey's HSD post hoc analysis ($P < 0.05$) (**b-e**, **h**). Exact *P* values are indicated above the bars (**a**) or provided in Supplementary Data 7 (**b-e**, **h**). Scale bars, 1 cm (**a-e**, **g**). Source data are provided as a Source Data file (**a-f**, **h**). All experiments were repeated at least two times with similar results.

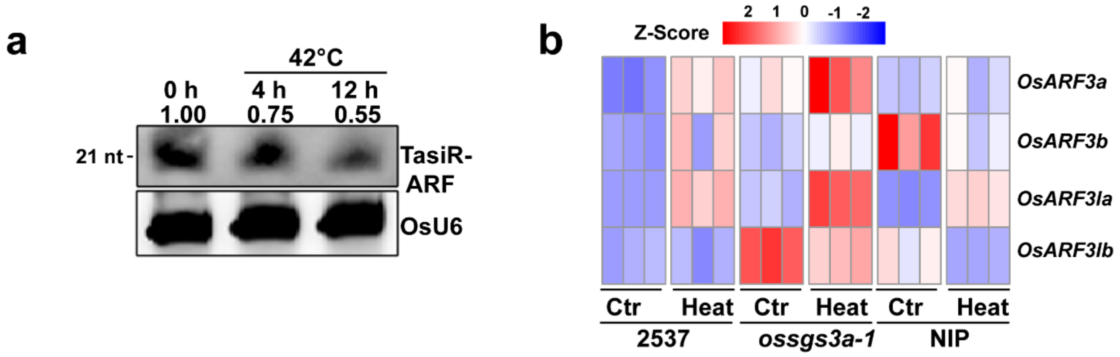

Supplementary Fig. 11 Heat stress down-regulated the abundance of tasiR-ARFs and up-regulated the expression of *OsARF3a* and *OsARF3la*.

a RNA blot of tasiR-ARFs in the leaves of 2-week-old NIP after heat treatment for 4 h and 12 h in a growth chamber. *OsU6* was used as a loading control. The intensity of the blots was quantified. Experiments were repeated two times with similar results. **b** Levels of *OsARF3a*, *OsARF3b*, *OsARF3la*, and *OsARF3lb* mRNAs in heat-stressed 2537, *ossgs3a-1*, and NIP. Data were extracted from transcriptomic analysis. Source data are provided as a Source Data file (**a-b**).

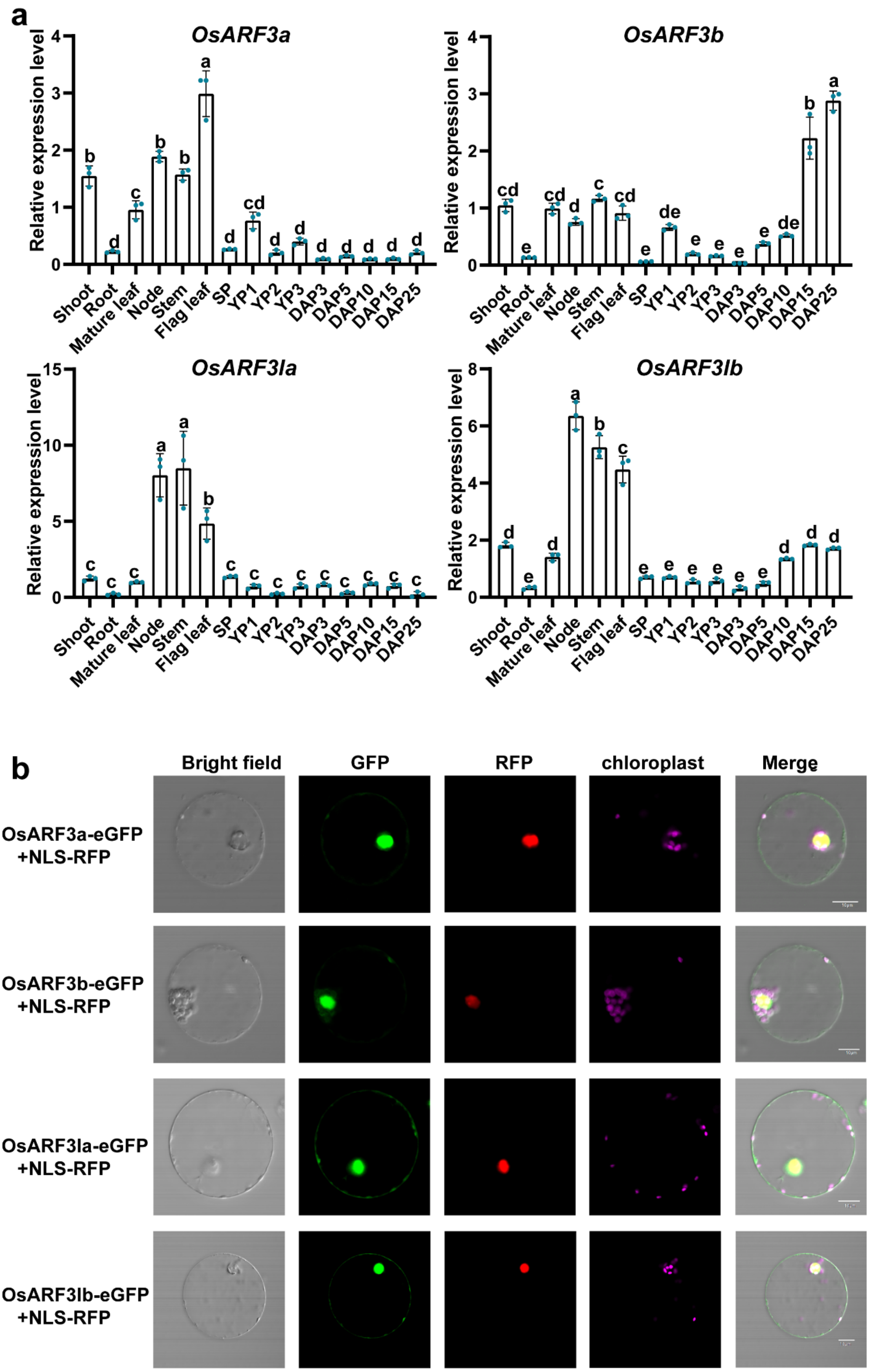

Supplementary Fig. 12 The expression pattern and subcellular localization of

***OsARF3a*, *OsARF3b*, *OsARF3la* and *OsARF3lb*.**

a Levels of *OsARF3a*, *OsARF3b*, *OsARF3la*, and *OsARF3lb* mRNAs as measured by qRT-PCR. The samples were same as those in Supplementary Fig. 2d. Data are presented as means ± s.d. (n = 3, biologically independent samples). *OsActin* served as an internal control. Different letters indicate significant differences according to one-way ANOVA with Tukey's HSD post hoc analysis ($P < 0.05$). Exact P values are provided in Supplementary Data 7. Source data are provided as a Source Data file. **b** Representative localization images of OsARF3a-eGFP, OsARF3b-eGFP, OsARF3la-eGFP, and OsARF3lb-eGFP transiently expressed in the NIP protoplast. NLS-RFP served as a nuclear marker. Scale bars, 10 μm. All experiments were repeated three times with similar results.

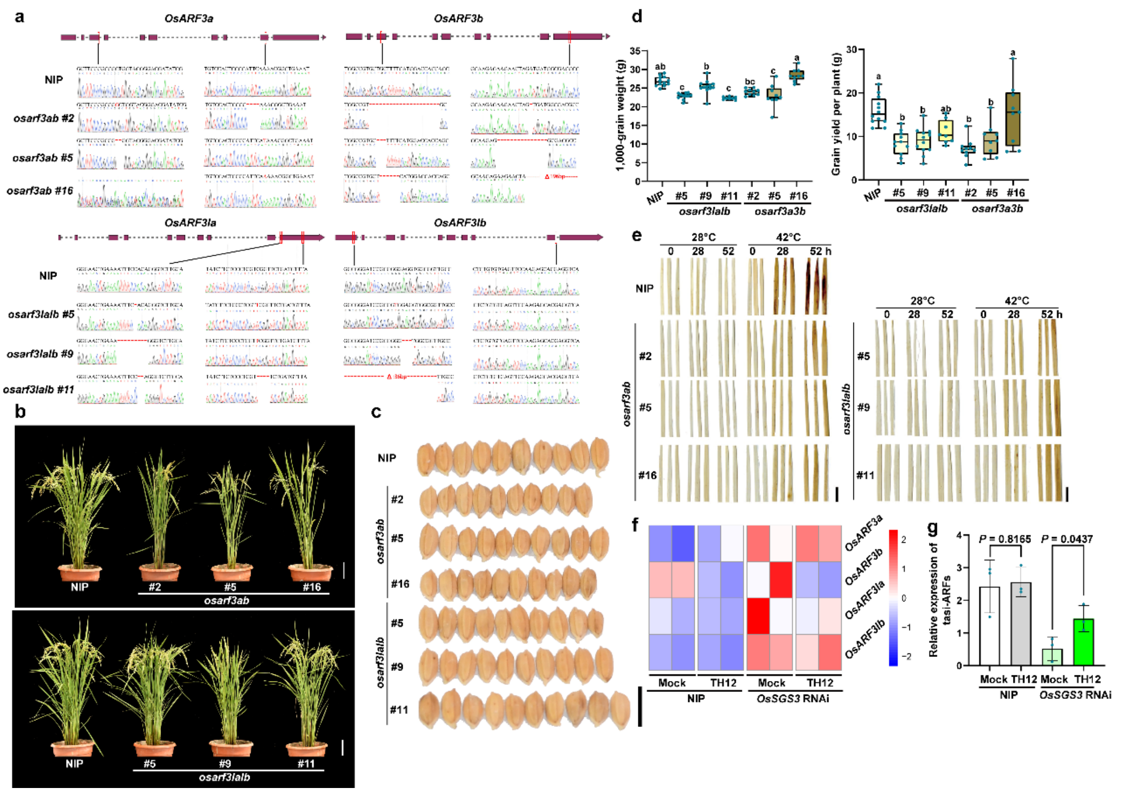

Supplementary Fig. 13 OsARF3a/3b/la/lb modulate disease resistance and thermotolerance in rice.

a Schematic of the mutation of *OsARF3a*, *OsARF3b*, *OsARF3la*, and *OsARF3lb* in *osarf3ab* or *osarf3lalb* mutants, respectively. Note that *osarf3ab* #15 and #16 carry the same mutation of *OsARF3a* and *OsARF3b*. **b, c** Plants (**b**) and grains (**c**) of NIP, *osarf3ab*, and *osarf3lalb* mutants grown under high field temperature. **d** The grain yield per plant and 1,000-grain weight of NIP, *osarf3ab*, and *osarf3lalb* mutants grown under high field temperature, shown as box plots ($n \geqslant 8$, biologically independent samples). Box plots show the median (central line) and interquartile range (IQR; from the 25th to 75th percentile); whiskers extend to minimum and maximum values within 1.5 times the IQR. Significant differences were determined by one-way ANOVA with Tukey's HSD post hoc analysis ($P < 0.05$). Exact P values are provided in Supplementary Data 7. **e** DAB staining revealed decreased accumulation of H_2O_2 in *osarf3ab* and *osarf3lalb* mutants. For heat stress, 12-day-old seedlings were treated at 42°C for 28 h or 52 h in a growth chamber. Experiments were repeated three times with similar results (**d**, **e**). Scale bars, 10 cm (**b**) or 1 cm (**c**, **e**). **f** Levels of *OsARF3la*, *OsARF3lb*, *OsARF3a*, and *OsARF3b* mRNAs in NIP and *OsSGS3* RNAi transgenic plants after inoculation with TH12 for 24 h. Data were extracted from transcriptomic analysis. **g** Levels of tasiR-ARFs in the leaves of NIP and *OsSGS3* RNAi transgenic plants after inoculation with TH12 for 24 h as determined by qRT-PCR. *OsU6* was used as an internal control. Data are means ± s.d. ($n = 3$, biologically independent samples). Significant differences were determined by two-tailed Student's *t*-test. Source data are provided as a Source Data file (**d**, **f**, **g**).

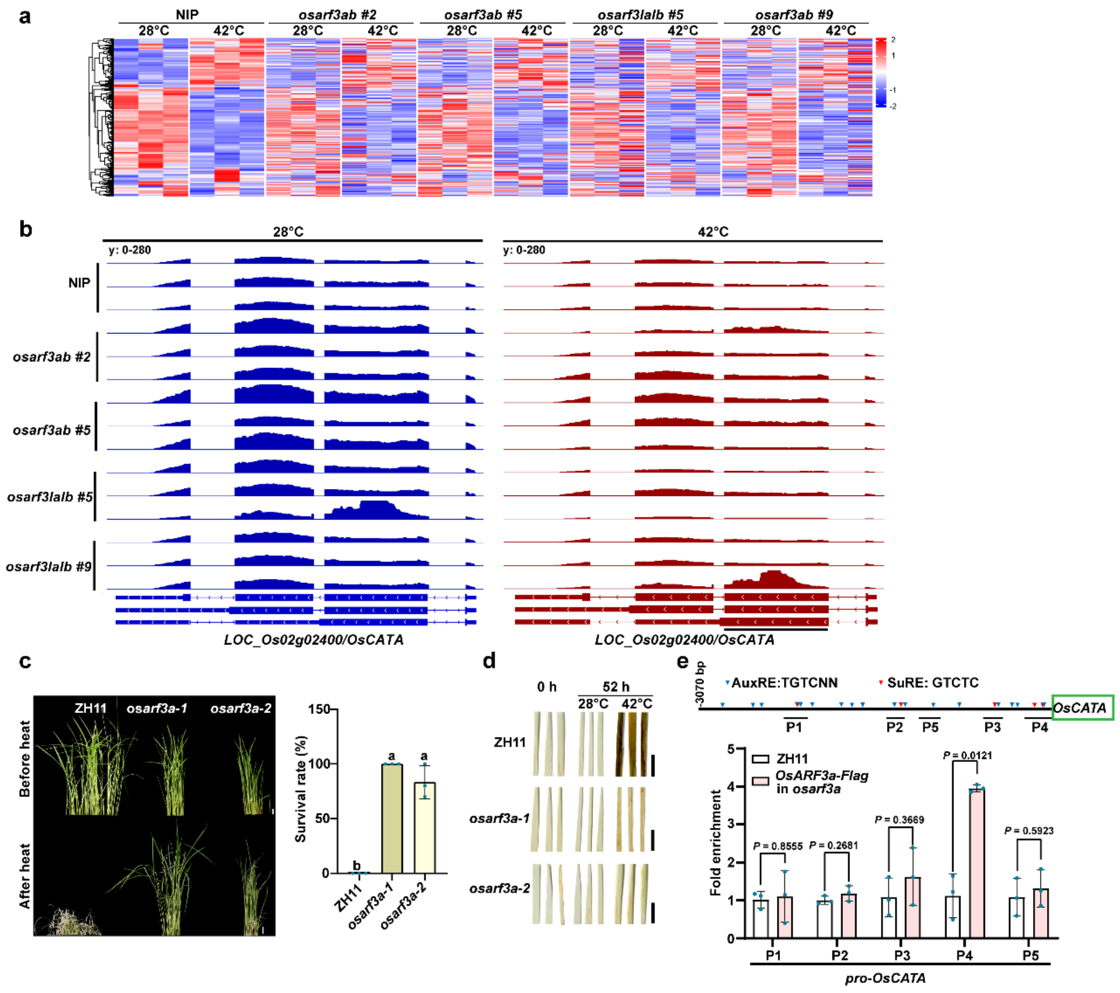

Supplementary Fig. 14 OsARF3a binds to the promoter of *OsCATA* and may modulates *OsCATA* expression.

a Transcriptomic analysis of ROS-related genes that are modulated by OsSGS3a in 2-week-old wild-type, *osarf3ab* and *osarf3lalb* knock-out mutants under heat stress. **b** Genome browser view of the *OsCATA* mRNAs in the leaves of NIP, *osarf3ab* and *osarf3lalb* knock-out mutants. Scale bar, 600 bp. **c** *osarf3a-1* and *osarf3a-2* mutants displayed enhanced thermotolerance than the wild-type ZH11. After heat stress treatment at 42°C for 52 hours, seedlings were recovered at 28°C for 2 days before photographed. The survival rate was shown as mean ± s.d. (n = 3, biologically independent samples). **d** DAB staining revealed decreased accumulation of H_2O_2 in *osarf3a-1* and *osarf3a-2* mutants. For heat stress, 12-day-old seedlings were treated at 42°C for 52 h in a growth chamber. Scale bars, 1 cm (**c**, **d**). **e** OsARF3a binds to the *OsCATA* promoter *in vivo*. 12-day-old ZH11 and *OsARF3a-Flag* in *osarf3a* seedlings were treated at 42°C for 6 h in a growth chamber and sampled for ChIP assay. Data are shown as relative fold enrichments over the background (ZH11). The locations of 18 AuxREs (Blue) and 5 SuREs (Red) are indicated by triangles above the gene model. The regions validated by ChIP-qPCR were marked by a bar below the gene model. Values are mean ± s.d. (n = 3, biologically independent samples). Significant differences were determined by one-way ANOVA with Tukey's HSD post hoc

analysis ($P < 0.05$) (**c**) or two-tailed Student's *t*-test (**e**). Exact *P* values are indicated above the bars (**e**) or provided in Supplementary Data 7 (**c**). Source data are provided as a Source Data file (**a**, **c**, **e**). Experiments were repeated two times with similar results (**c-e**).
