# Supplementary material for: The OsSGS3-tasiRNA-OsARF3 module orchestrates abiotic-biotic stress response trade-off in rice
Source: Nat Commun. 2023 Jul 24;14:4441. doi: 10.1038/s41467-023-40176-2 (PMC10366173; doi:10.1038/s41467-023-40176-2)
Supplement: Supplementary file 3 — Description of Additional Supplementary Files [file 41467_2023_40176_MOESM3_ESM.pdf]

## Description of Additional Supplementary Files

**Supplementary Data 1**. Daily temperature in Shanghai and Hainan

**Supplementary Data 2**. The results of LC-MS/MS analysis.

**Supplementary Data 3**. Summary of RNA-seq and small RNA profiles generated in this study.

**Supplementary Data 4**. Heat-related DGEs in heat-stressed 2537, *ossgs3a-1*, NIP, *osarf3ab* and *osarf3lalb* knock-out mutants.

**Supplementary Data 5**. Immunity-related DGEs in NIP and *OsSGS3* RNAi transgenic plants after inoculation with TH12 for 24 h.

**Supplementary Data 6**. Primers used in this study.

**Supplementary Data 7**. Statistical summary—a summary of all statistical analysis by ANOVA.
